# Advances in Plant–Pathogen Interaction: New Challenges for Sustainable Disease Management

**DOI:** 10.3390/biology12020203

**Published:** 2023-01-28

**Authors:** Maria Doroteia Campos, Maria do Rosário Félix

**Affiliations:** 1 MED—Mediterranean Institute for Agriculture, Environment and Development & CHANGE—Global Change and Sustainability Institute, Institute for Advanced Studies and Research, Universidade de Évora, Pólo da Mitra, Ap. 94, 7006-554 Évora, Portugal; 2 MED—Mediterranean Institute for Agriculture, Environment and Development & CHANGE—Global Change and Sustainability Institute, Departamento de Fitotecnia, Escola de Ciências e Tecnologia, Universidade de Évora, Pólo da Mitra, Ap. 94, 7006-554 Évora, Portugal

## 1. Introduction

Plant pathogens cause huge losses and have been an important constraint to a worldwide increase in crop production and productivity. They are aggravated by agricultural intensification and monocultures, leading to new emergent diseases. Therefore, relevant efforts have been made in the search for environmentally friendly biocontrol agents, especially after UE directories for commercialization withdrew several chemical substances used for pest and disease control and new rules were established to reduce agricultural greenhouse gas emissions in the 2015 Paris Agreement of the United Nations Framework Convention on Climate Change.

The European Union, FAO, and the United Nations largely promote and finance projects and programs to introduce crop protection principles that can attain sustainable agriculture [1]. However, developing sustainable plant protection strategies requires new insights into the biology and evolution of the corresponding pathogens and the use of new technologies to augment the efficacy of disease control, bringing new challenges to agriculture production to feed a continuously growing world population. Appropriate management practices and biological control strategies using antagonistic microorganisms may constitute alternative treatments to control plant pathogens, with quite promising prospects for several plant species and pathogens [2,3]. Additionally, the identification of plant key functional genes involved in plant–pathogen interaction, as well as the understanding of the molecular basis of compatible interactions during pathogen attack, offer great opportunities to apply genetic engineering efforts for incorporation of new sources of resistance for plant protection against pathogens [4,5].

## 2. Special Issue Overview

The Special Issue of *Biology,* “Plant–Pathogen Interaction 2.0”, includes two original research articles and four reviews focusing on different aspects of plant–pathogen interaction, especially in agriculture systems, and the protection of plants against pathogens.

As mentioned above, identifying candidate genes in susceptible and resistant responses may facilitate genetic engineering efforts to incorporate new sources of plant resistance/tolerance against pathogens for sustainable plant disease management. In the scope of this subject, Campos et al. [6] highlight the importance of genes encoding transcription factors (TFs) in tomato response to pathogens, focusing on different families of TFs selected for their abundance, importance, and availability of functionally well-characterized members in response to pathogen attack. These authors review tomato TFs’ roles and possibilities related to their use for engineering pathogen resistance in tomato in response to a broad range of pathogens. Focusing on a specific target gene, the work developed by Zhao et al. [7] characterizes and functionally analyzes the *Triticum aestivum* macrophage migration inhibitory factor (*Ta*MIF1), whose function was still unknown in plants. These authors report upregulation of *TaMIF1* to *Puccinia striiformis* f. sp. *Tritici* (*Pst*) infection of wheat, with functional analysis revealing that *Ta*MIF1 was capable of suppressing programmed cell death, indicating its role in plant immunity, whilst silencing *Ta*MIF1 decreased the susceptibility of wheat to *Pst*. The review performed by Rusinque et al. [8] also highlights the importance of genomic tools in understanding the molecular responses of plants to infection by the nematode *Meloidogyne graminicola*, with transcriptomic analysis identifying several key genes in the infection process.

Furthermore, Rusinque et al. [8] focus their review on the distribution, biology, identification, and management of the nematode *M. graminicola,* considering the impact that this pathogen has on agricultural systems, especially rice production. The review from Bharudin et al. [9] reports the search for alternative methods to protect palm oil against a devastating disease predominantly caused by the fungus *Ganoderma boninens*, pointing to the importance of methods used in disease management that include cultural practices, chemical treatments, and antagonistic microorganism manipulations. The authors also propose future directions to increase the understanding of fungus invasion mechanisms against oil palm. The use of biological control strategies using antagonistic microorganisms as an alternative treatment to control diseases is also reflected by the research performed by Degani et al. [10]. These authors identify an isolate of *Trichoderma asperellum* with potential antifungal activity against *Magnaporthiopsis maydis*, a fungus responsible for a destructive vascular disease of maize.

Finally, an important contribution to the search for alternatives to synthetic agrochemicals is given by Zobir et al. [11]. Looking for alternatives to the higher amount of agrochemical usage to meet the gaps between food production and consumption, with conventional agro-nanochemicals presenting reduced effectiveness of the active ingredient in reaching the target that affects the environment and life, these authors provide an overview of the current status of the host–guest supramolecular chemistry of nanopesticides to combat various pests for plant protection.

In this context, the Special Issue “Plant–Pathogen Interaction 2.0” introduces new advances to unravel the complexity of plant–pathogen interactions, with a special focus on the search for sustainable methods to protect plants against pathogens.

We would like to thank all the authors for their papers submitted to this Special Issue. We would also like to acknowledge all the reviewers for their careful and timely reviews to help improve the quality of this Special Issue, as well as the staff at the editorial office of *Biology* for their efficient assistance.
